# New estimation of the prevalence of chronic pulmonary aspergillosis (CPA) related to pulmonary TB – a revised burden for India

**DOI:** 10.1016/j.ijregi.2022.11.005

**Published:** 2022-11-18

**Authors:** David W. Denning, Donald C. Cole, Animesh Ray

**Affiliations:** 1 Manchester Fungal Infection Group, Faculty of Biology, Medicine and Health, Manchester Academic Health Science Centre, University of Manchester, Manchester, UK; 2 Global Action for Fungal Infections, Geneva, Switzerland; 3 Dalla Lana School of Public Health, University of Toronto, Toronto, Ontario, Canada; 4 Department of Medicine, All India Institute of Medical Sciences, New Delhi, India

**Keywords:** *Aspergillus*, tuberculosis, itraconazole, voriconazole, lobectomy, survival

## Abstract

**Background:**

Chronic pulmonary aspergillosis (CPA) may be confused with, or a coinfection of, pulmonary tuberculosis (PTB), or may manifest itself after completion of antituberculous therapy (ATT).

**Methods:**

Literature searches were conducted on PubMed. The selected studies stated the timing of CPA diagnosis with respect to PTB. The key assumptions for estimating the annual incidence, annual deaths, and 5-year-period prevalence related to CPA were: of the clinically diagnosed PTB patients , 19% of those HIV-negative had CPA and 7% of HIV-positive patients had CPA; the percentage of patients presenting in the first year after PTB diagnosis or developing CPA as ATT finished was 10%; the annual rate of development of CPA from 2–5 years after PTB diagnosis was 1.5%; and the mortality of CPA was 20% in year 1 and 7.5% thereafter to year 5.

**Findings:**

In India, the annual incidence of CPA arising in PTB patients in 2019 was estimated to be 363 601  cases (range 254 521 - 472 682) and 42 766 deaths (range 29  936–55 595) — 10.5% of total PTB deaths. The 5-year-period prevalence of CPA was estimated at   1 575 716 , with an additional 100 715 deaths' total range of deaths 100 436– 186 525) annually.

**Interpretation:**

The revised estimation indicates a substantial unmet need for better diagnosis of CPA as part of a complex PTB-related respiratory morbidity burden.

## Highlights

• CPA can occur concurrently with, and be mistaken for, TB; it can also appear after treatment of pulmonary TB.
• A re-estimation of the prevalence of CPA was found to be around 1.5 million.
• Over 42 000 deaths occur within 12 months of misdiagnosis of pulmonary TB.
• Around 100 000 deaths occur annually from CPA presenting years after TB cure.
• Detection of *Aspergillus* IgG antibody and high-volume sputum fungal culture is necessary.

## Introduction

Post-tuberculous pulmonary complications are receiving greater attention. Chronic pulmonary aspergillosis (CPA) is one of the most serious of these. The term CPA was introduced in 2003 as a catch-all for chronic cavitary, chronic fibrosing, and the older term chronic necrotizing pulmonary aspergillosis, as well as simple and complex aspergilloma (Denning et al., 2003). The majority of cases diagnosed have chronic cavitary pulmonary aspergillosis, especially linked with pulmonary tuberculosis (PTB) and non-tuberculous mycobacterial infection (NTM). The diagnosis of CPA rests on chronic symptoms (usually > 3 months) in a non-immunocompromised patient, characteristic radiological features (pleural thickening, cavitation, and pericavitary infiltrates with or with a fungal ball), and microbiological evidence of *Aspergillus* infection — usually a positive *Aspergillus* IgG antibody test (see Supplementary Table 1 for diagnostic details of each study). The details and challenges of making a diagnosis of CPA have recently been reviewed (Denning, 2021).

**Figure 1:**
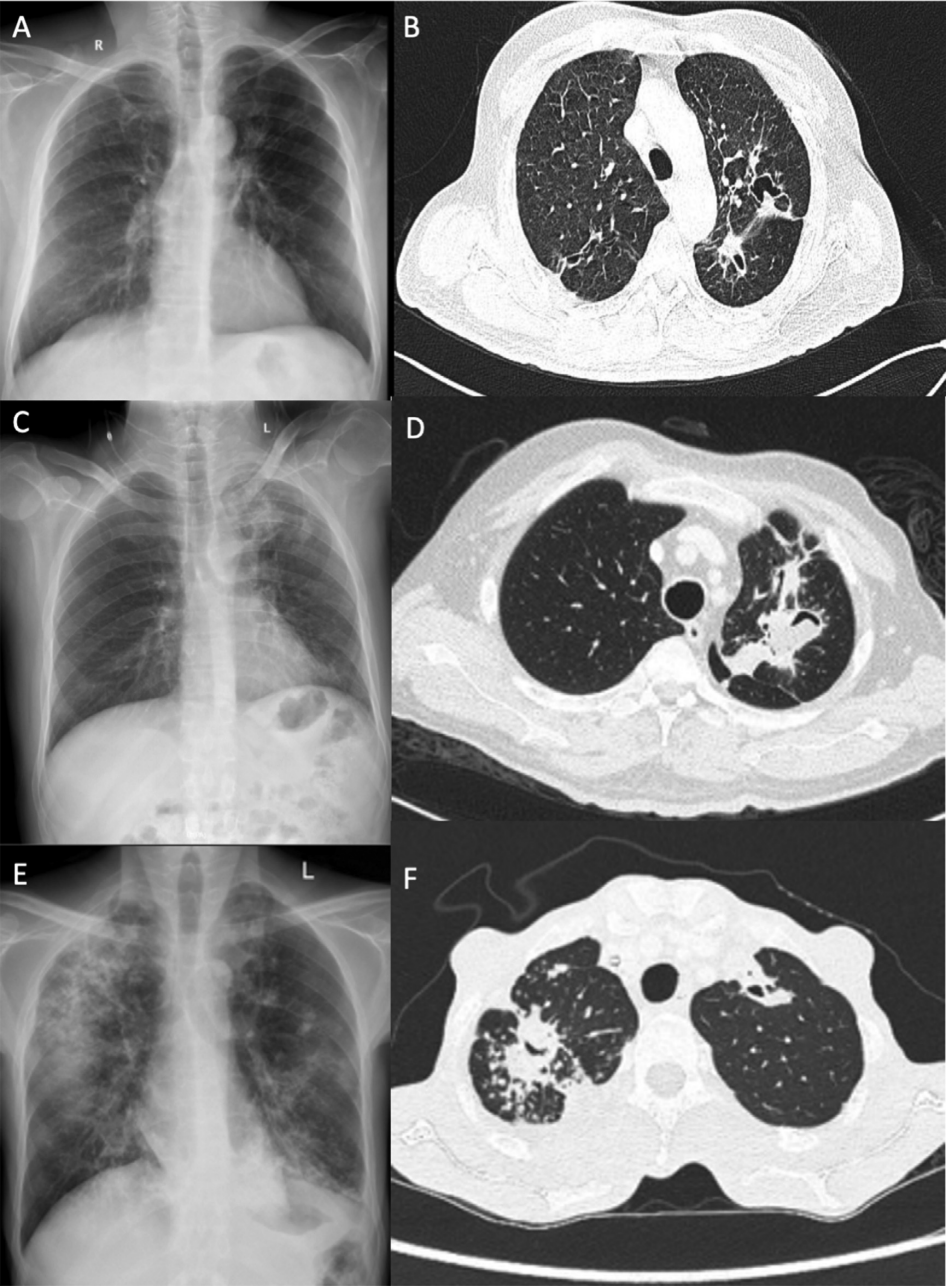
Case 1: 50-year-old male with symptoms of chronic productive cough. (A) Chest radiograph showed reticular opacities in the right upper zone of the lung. (B) Chest CT showed intrabronchial aspergilloma in the left upper lobe. Subsequent tests for IgG against Aspergillus were positive and a diagnosis of CPA was made. Diagnosis of CPA hinges on combined clinico-radio-microbiological features. Case 2: 45-year-old male with past history of tuberculosis presenting with complaints of intermittent hemoptysis. (C) Chest radiograph showed heterogenous opacities in the left upper zone. (D) Chest CT showed air crescent cavity signs in the left upper lobe, suggestive of aspergilloma. Chest radiograph features may be non-specific and advanced chest imaging may be required. Case 3: 30-year-old male with fever and weight loss over a 4-month period. (E) Chest radiograph showed bilateral reticulonodular opacities in the lung, along with consolidation in the right upper and middle zone. (F) Chest CT showed right and left upper lobe cavities, with pleural thickening. Workup for CPA was positive. Presence of pleural thickening is an important marker of the presence of CPA.

In the late 1960s, the Research Committee of the British Thoracic and Tuberculosis Association assessed nearly 500 patients cured of pulmonary PTB but who had a residual cavity, and found 25% with detectable *Aspergillus* precipitins in blood. Both precipitins and radiological features of an aspergilloma were detectable in 14% of patients at 12 months and in 22% at 3–4 years (Anonymous, 1968; Anonymous, 1970). In 2011 Denning and colleagues applied these estimates to global and country PTB survivors from 2007 (Denning et al., 2011). The authors commented: ‘CPA is a sequel of PTB…can account for progressive lung destruction and the persistence of symptoms after successful antituberculous treatment (ATT) and can mimic smear-negative PTB.’ Two recent papers from Vietnam and India reported that 54-57% of patients with recurrent symptoms after PTB treatment completion had CPA (Nguyen et al., 2021; Singla et al., 2021) (Figure 1 shows three cases of CPA with varied presentations).

Estimates of CPA for India — 92 042 annual cases and a 5-year-period prevalence of 290 147 — were published in 2014 (Agarwal et al., 2014). Their study omitted three groups of patients: 1) misdiagnosed PTB (i.e. clinically diagnosed, laboratory negative cases) (Kwizera et al., 2021); 2) those with dual infection; and 3) those who developed CPA at the end of their ATT therapy or immediately thereafter, were operated upon, or died. Our study aimed to update our 2011 approach by: a) including studies of CPA published since 2011; b) explicitly laying out the bases for our estimates of proportions at each step of our estimation approach, including these additional groups of patients; and c) applying this approach to estimating CPA incidence and mortality in India, the country with the world's largest PTB incidence.

## Methods

### Literature searches

Epidemiological (clinical and population) literature on CPA has been continuously tracked since 2010, based on weekly automated searches through the US National Library of Medicine. These data inputs were supplemented with comprehensive searches unrestricted by geography (Supplementary material 1–5). No ethical permission was required or sought for the research, since no new patient data were acquired. Series were required to have clearcut definitions of CPA, including symptomology, radiology, and mycological evidence, unless an aspergilloma was present on imaging. Culture and *Aspergillus* antibodies alone were not sufficient for the diagnosis.

#### Estimation approach

This global literature was drawn upon to develop a staged estimation approach over six steps:

1) The proportion of newly presenting patients who may have been misdiagnosed as having PTB.
2) The proportion of newly presenting patients who may have had CPA and PTB (coinfection).
3) The proportion of PTB survivors who developed CPA on ATT completion and presented clinically within 12 months of starting ATT.
4) The proportion of these two groups who died.
5) The proportion of PTB survivors without initial CPA who presented each year with CPA in years 2–5 after initiating ATT.
6) The proportion of these later cases of CPA who died.

For each of these steps, commentary on the heterogeneity in the literature is provided. In setting forth arguments for our best estimate of the relevant proportions, our study relied more heavily on some key papers (Table 1). In view of diagnostic uncertainty early in the course of TB therapy, a sensitivity range of ± 30% was added to the incidence figures and to early deaths.

**Table 1:** Key assumptions for estimations, all pertaining to only pulmonary PTB, and key sources. Data are taken from multiple countries and applied to India.

Data point or variable	Location (source)	Design and diagnosis	Generic
Pulmonary aspergillosis in India	India (WHO, 2019)	Estimated incidence, based on 79% of all cases being pulmonary	2 059 200
Percentage with cavitation at end of therapy	Multiple (Meghji et al., 2016)	Not applicable	22%
Coinfection of confirmed PTB with CPA, HIV negative	Indonesia (Setianingrum et al., 2022)	Cross-sectional, 4–6 months after starting PTB therapy, *Aspergillus* IgG antibody, symptoms, chest X-ray and some CT scans	3%
Coinfection of confirmed PTB with CPA, HIV positive	Nigeria (Oladele et al., 2017)	*Aspergillus* IgG antibody, symptoms, chest X-ray	7%
CPA among clinically diagnosed PTB, HIV negative	Nigeria (Oladele et al., 2017)	*Aspergillus* IgG antibody, symptoms, chest X-ray	19%
CPA among clinically diagnosed PTB, HIV positive	Nigeria (Oladele et al., 2017)	*Aspergillus* IgG antibody, symptoms, chest X-ray	10%
CPA at end of PTB therapy 6–12 months after diagnosis	Indonesia (Setianingrum et al., 2020) Indonesia, (Setianingrum et al., 2022)	Cross-sectional, 4–6 months after starting PTB therapy and prospective, pulmonary PTB patients at beginning and end of therapy *Aspergillus* IgG antibody, symptoms, chest X-ray and some CT scans	10% (8–13%)
Mortality of CPA, first 12 months	Multiple (see Table S2 and Figure 2)	Cohort studies, with multiple diagnostic modalities	20%
Mortality of CPA, 2–5 years	Multiple* (see Table S2 and Figure 2)	*Aspergillus* IgG antibody, symptoms, chest X-ray and CT scans	7.5%
CPA development, 1–2 years after PTB cure	South Korea (Kim et al., 2022)	*Aspergillus* IgG antibody, symptoms, chest X-ray and CT scans	3%, up to year 2; 7.5% over 5 years
CPA development, 2–5 years after PTB cure	UK (Anonymous, 1968 and 1970) Uganda (Page, 2019)	Prospective, *Aspergillus* precipitins, aspergilloma visible on chest X-ray Cross-sectional and prospective, *Aspergillus* IgG antibody, symptoms, chest X-ray and CT scans	6.5% or 0.2% annually, with or without cavitation

WHO = World Health Organization

⁎ Survival data from the UK, the USA, Japan, South Korea, Italy, Brazil, Hong Kong, France, Spain, Pakistan, and Australia

#### CPA misdiagnosed as PTB (step 1)

There have been many reports of CPA initially misdiagnosed as tuberculosis (yet usually treated with ATT). In Lagos, a cross-sectional survey of patients with clinically diagnosed pulmonary PTB (smear and GeneXpert negative in most patients) found that 10% of HIV-infected and 19% of HIV-negative people had CPA, based on chest radiographs (not CT scan) (Oladele et al., 2017). Some patients were *Aspergillus* antibody negative and/or asymptomatic, but had evidence of an aspergilloma — these cases were excluded in our estimates. In Manaus, a cross-sectional study of 213 smear-negative ‘PTB’ patients found 15 with a pulmonary mycosis based on culture, serology, and CT scanning, of whom 10 (4.7%) had CPA (Matsuda et al., 2021). In Uzbekistan, 12 (20%) of 60 smear-negative TB patients had CPA (Toychiev et al., 2022).

#### CPA and PTB coinfection (step 2)

Dual infection has also been reported. In Pakistan, Iqbal et al. (2016) found nine (13%) of 69 patients with dual infection, but it was not clear at what point in their PTB treatment course CPA was diagnosed (Iqbal et al., 2021). Likewise, in Iran, nine of 94 (10%) of patients had CPA during or immediately after ATT therapy (Hedayati et al., 2015). In Uzbekistan, 16 (11.4%) of 140 patients with smear-positive TB had CPA (Toychiev et al., 2022). The lack of precise timing of CPA relative to initiation of ATT prevented the use of these studies in our estimates. In Nigeria, 2 (7%) of 27 HIV-positive patients and 1 (6%) of 18 HIV-negative patients with confirmed PTB diagnosis had features of CPA (Oladele et al., 2017). In Jakarta, a prospective study of 216 patients starting ATT (42% laboratory confirmed) found CPA in 14 (7.9%) overall (Setianingrum et al., 2022). Our study assumed PTB-CPA coinfection rates of 3% and 6% in HIV-negative and HIV-positive patients, respectively, during the early phase of ATT therapy.

#### CPA developing after ATT (step 4)

A significant minority of patients develop CPA towards the end of ATT. In Jakarta, at the end of their ATT therapy, 128 of the original 216 patients were followed up (Setianingrum et al., 2022). Features of CPA resolved in nine patients and persisted in three, and an additional 12 patients (11.7%) developed CPA. A cross-sectional study, also in Indonesia, included an additional 71 patients assessed at the end of their ATT therapy and found that 13% had evidence of CPA (Setianingrum et al., 2020). Those patients who develop CPA during and soon after competing ATT are assumed to have a 20% mortality and die the same year. This may slightly overestimate the number of deaths in that year, and thus slightly underestimate the number of deaths in year 2.

#### CPA developing years after tuberculosis cure (step 5)

A proportion of patients cured of PTB develop CPA. A meta-analysis of post-TB radiological abnormalities found percentages with residual cavitation on chest radiographs ranging from 8.3% to 83.7% and on CT imaging from 7.4% to 36.6% (Meghji et al., 2016). Pleural thickening of > 10 mm, a highly characteristic feature of CPA, was found in 19.6–46.0% of patients. Aspergilloma or fungal ball was not documented. Our study used a mean rate of 22% in our estimates, as previously (Denning et al., 2011).

A prospective study in Gulu, Uganda over 2 years, in both HIV-positive and HIV-negative survivors of PTB, found the annual rate of development of CPA to be 6.5% in those with residual cavitation and 0.2% in those without (a mean of 4.9-6.3%) 2–7 years after ATT (Page et al., 2019). There was a non-significant difference between HIV-positive and HIV-negative patients, with a higher loss to follow up in the former. There are very few data on occurrence and survival beyond 5 years, which is why our study did not attempt to extend the estimate beyond this time. As a result, our prevalence and mortality estimates are likely to be slightly lower than the true values.

As an alternative approach to modeling post-TB CPA, our study utilized overall annual rates of CPA development, regardless of cavitation. In Charleston, USA 14 of 286 (4.9%) patients treated for PTB had a subsequent discharge radiological diagnosis of aspergilloma over the following 8 years (Tomlinson and Sahn, 1987). Note that an aspergilloma is visible on chest X-ray in about 20% of patients with CPA (Nguyen et al., 2021). CPA, usually with an aspergilloma, was found in 8.3% of 350 previously treated PTB patients who were subsequently hospitalized in Rio de Janeiro, Brazil over an unclear time frame (Ferreira-Da-Cruz et al., 1988). A prospective study in Seoul, South Korea found 3% of culture-proven PTB cases to have CPA in the 2 years after completion of ATT (Kim et al., 2022). For consistency, our study used 1.5% annually, and modeled the overall 5-year-period prevalence of CPA after TB using 7.5%.

#### Mortality from CPA (steps 3 and 6)

**Figure 2:**
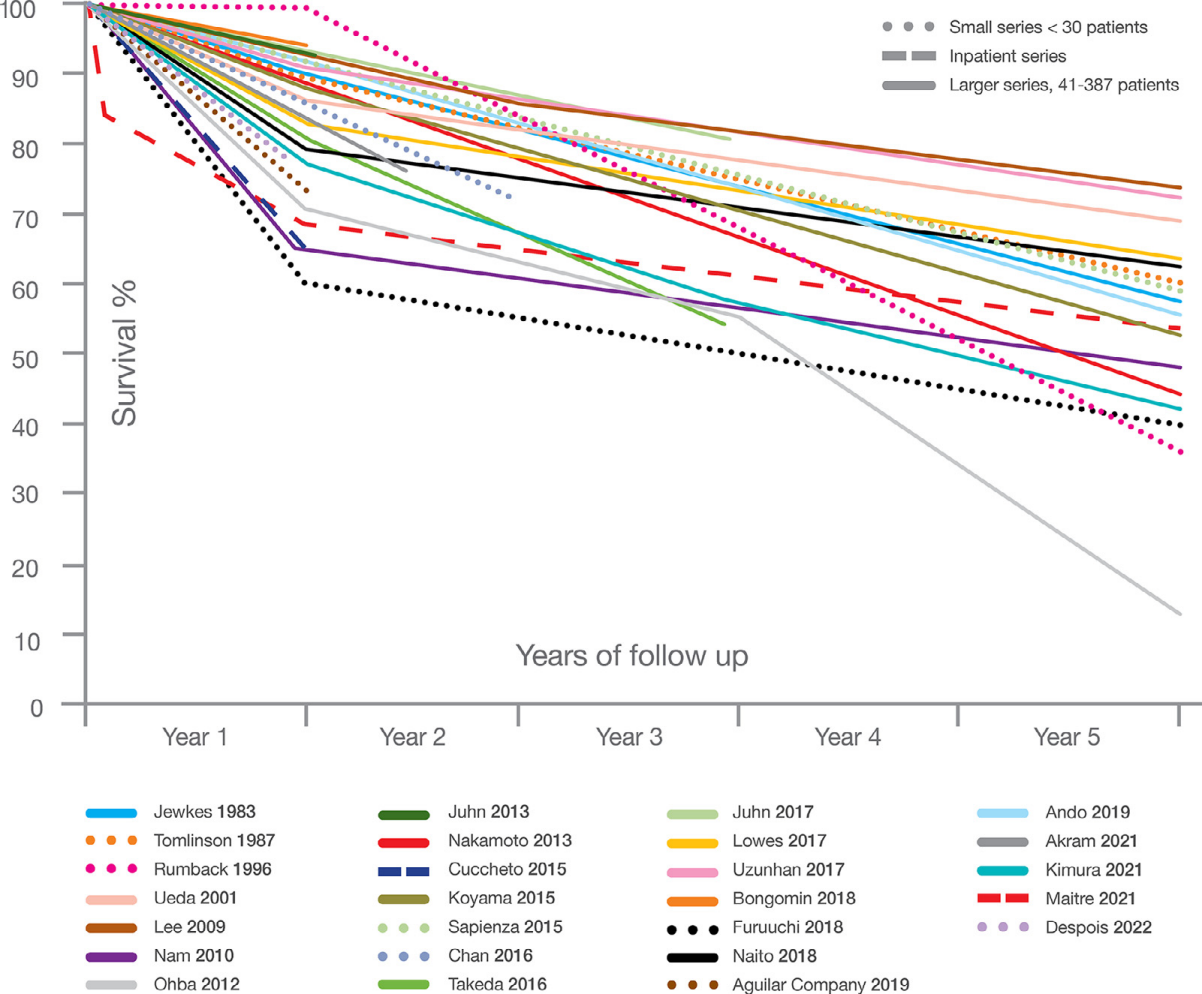
CPA survival curves from different primary studies. Solid lines represent larger, mostly outpatient cohorts; dotted lines represent smaller studies; and the two with dashed lines represent inpatient cohorts.

The early stages of CPA are usually asymptomatic. CPA is usually progressive, but not always, with substantial variation in different patients. For example, bilateral CPA progresses faster than unilateral disease, and those with bilateral aspergillomas do particularly badly (Lowes et al., 2017). Figure 2 shows a collation of cohort studies that reported total mortality up to 5 years (Table S2). Most CPA patients had one or more underlying conditions, usually comorbidity affecting the lungs, but also diabetes, HIV, or autoimmune disorders.

For estimation purposes our study assumed a 20% first-year mortality from diagnosis, including misdiagnosis of PTB or dual infection (step 3), and 7.5% annually thereafter (step 4), giving an overall 50% mortality during a 5-year period.

#### Application of CPA burden estimation approach to India

These best estimates were then combined in an Excel spreadsheet and applied to India's 2019 PTB data (World Health Organization, n.d.), used retrospectively for years 2016–2019 inclusive to compute the 5-year-period prevalence of CPA presenting years after completion of ATT therapy. As PTB numbers fell in India (until 2020), this provided a conservative estimate.

## Results

India's PTB incidence was estimated by WHO using a well-described combination of reported cases and imputation of missing cases from prevalence and incidence surveys (Glaziou et al., 2018). In 2019, TB incidence was 2 640 000 (rate 188/100 000), of which 79% were pulmonary. There were an estimated 42 000 HIV-positive patients with PTB. Among case notifications, including both new and relapsed cases, 79% were pulmonary, 54% of which were bacteriologically confirmed. Only 18% of cases were tested with rapid diagnostics. Among the estimated number of PTB patients, 394 000 (19.4%) of HIV-negative patients and 8800 (20.8%) of HIV-positive patients died. It was assumed that mortality was proportionately attributable to the incidence of pulmonary and non-pulmonary TB.

**Figure 3:**
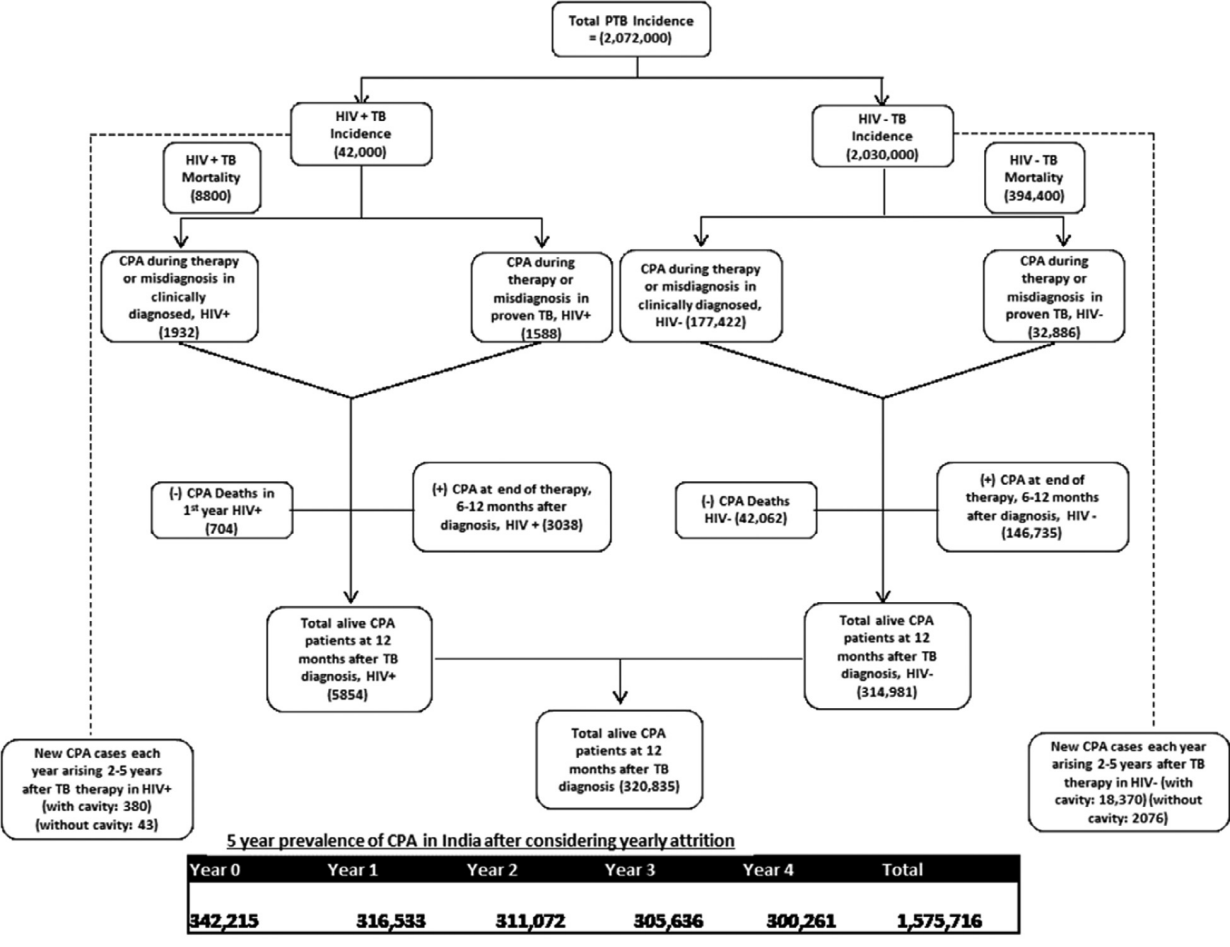
Distribution of CPA cases in HIV-infected and non-HIV-infected patients at different time points.

Thus, the annual incidence of CPA arising in PTB patients (confirmed or suspected) in India in 2019 was estimated to be the sum of dual (or coinfection) cases and misdiagnoses at the beginning of therapy, giving -  213 828cases (30% sensitivity range of  149 679– 277 976) (Table 2 and Figure 3). In addition, a further 149 774 cases arose during the few months after ATT completion. The total of 363 601 cases (range 254 521 – 472 682) was 17.5% of the total PTB cases presenting for care. Among these, an anticipated 42 766 cases died in that first year (range 29 936– 55 590), 10.6% of the total PTB deaths.

**Table 2:** Estimates of the incident cases of CPA in India, and associated mortality.

Sub-groups	Sub-group population estimate	CPA	Assumptions for CPA cases and deaths
PTB cases and CPA from (mis)diagnosis and during therapy (steps 1and2)			
HIV+, clinically diagnosed	19 320	1 932	10%
HIV−, clinically diagnosed	933800	176 422	19%
HIV+, proven PTB	22 680	1 588	7%
HIV−, proven PTB	1 096200	32 828	3%
**Total CPA cases**		**3** 213 828	
Deaths from CPA during and immediately after therapy for PTB (step 3)			
HIV+		704	20% CPA mortality over the 12 months after PTB diagnosis
HIV−		42 062	20% CPA mortality over the 12 months after PTB diagnosis
**Total CPA deaths in 12 months from PTB diagnosis**		**42** 766	
PTB cases and new CPA diagnoses 6–12 months after PTB diagnosis (step 4)			
HIV+	30 380	3 038	10%
HIV−	1 467 350	146 735	10%
**Total**		149 774	
Diagnosed CPA patients in 12 months after PTB diagnosis, including CPA deaths (steps 1,2,3and4)			
HIV+		6 558	
HIV−		357 043	
**Total**		363 601	
New CPA cases each year, arising 2–5 years after PTB therapy from this annual cohort of PTB cases (step 5)			
HIV+, with cavitation	6 016	391	6.5%
HIV+, without cavitation	21 109	43	0.2%
HIV−, with cavitation		18 885	6.5%
HIV−, without cavitation	1 030 082		0.2%
**Total**		21 379

The 5-year-period prevalence was calculated by taking the annual incidence, less the deaths (20%) in that year, and the deaths in the following year (7.5%), and adding the new cases arising in years 2, 3, 4, and 5, less their deaths, based on cavitation rates. This gave an anticipated 21 211 new cases each year, based on estimated incidence, in addition to the remaining CPA cases still alive from previous years. Our resulting estimate of CPA 5-year prevalence was 1 575 716 cases, while that of annual CPA deaths was 143 481 (range 100 436– 186 525). An alternative estimation of 5-year-period prevalence, which ignored post-ATT cavitation and assumed a 1.5% annual CPA rate, yielded remarkably similar results, with 21 379 new cases using cavitation rates and 20 219 using a flat annual CPA rate. In this case, the overall 5-year-period prevalence would fall from   1 575 716 to   1 561 527 (a 0.9% difference).

## Discussion

Our new estimate of prevalence was substantially larger than the previous figure – from 290 000 to 1.5 million. This was attributable to several factors. First was the inclusion of misdiagnosed (‘smear-negative’) cases, dual infections, and early infections during ATT and just after. Previously a flat 15% annual mortality was used, whereas for this study a rate of 20% in year 1, falling to 7.5% annually thereafter, was applied. The number of patients developing CPA after TB (years 2–5) was similar.

Our estimation approach focused on CPA related to PTB, ignoring all other underlying conditions for CPA (Anan et al., 2021). It is not discernible from the current state of knowledge whether in India there are patients with CPA who are not suspected of having TB, but could be diagnosed with CPA. In most large series from other countries (Anan et al., 2021) up to 80% of CPA patients had a history of, or concurrent, PTB.

The publication of country PTB estimates derives from both multiple sources and differing estimation approaches (Glaziou et al., 2018). Given the variability of current estimates, our study presents sensitivity analyses for CPA occurring early during the course of TB, combined with misdiagnoses. The low percentage of confirmed pulmonary PTB (56%) includes smear- and GeneXpert-negative cases, and cases without a sample obtained or analysed, which could have a significant impact on case numbers. Our study also presents a sensitivity analysis for early (and total) deaths, given the substantial variation in mortality rates over time in different cohorts (Figure 2), reflecting many factors that may or may not pertain to India. There are not enough large studies to permit robust range or distribution estimates like those that might be used in stochastic modeling approaches.

CPA diagnosis in prior studies has been dependent on *Aspergillus* antibody testing, which is at best 92% sensitive and usually less (Anan et al., 2021). In India, many CPA cases are caused by *A. flavus* (Sehgal et al, 2018). Performance of *A. fumigatus* antibody testing in these cases has not been fully evaluated (Denning, 2021). IgG antibody-negative cases can be diagnosed using a combination of characteristic imaging techniques, which show a fungal ball or other intracavitary features typical of CPA, together with positive culture or *Aspergillus* antigen detected in serum or BAL fluid.

Anti-*Aspergillus* IgG antibody is also not specific for CPA. Detectable antibodies are also found in those recovering from invasive aspergillosis, chronic invasive *Aspergillus* rhinosinusitis or granulomatous sinusitis, allergic bronchopulmonary aspergillosis, and *Aspergillus* bronchitis (usually seen in those with bronchiectasis), as well as some healthy people (Kim et al., 2022). In healthy populations, *Aspergillus* IgG was detectable in one of 222 (0.5%) in France (Guitard et al., 2012), 11 of 90 (12%) in Indonesia (Setianingrum et al., 2020), six of 42 (14%) in Belgium (Van Hoeyveld et al., 2006), and four of 150 (2.7%) in the UK (Wilopo et al., 2020). In patients with other respiratory conditions in whom aspergillosis had been ruled out, *Aspergillus* IgG was detectable in 14 or 16 (depending on the assay) of 192 (7.3–8.3%) in France (Dumollard et al., 2016), and 15 of 100 (15%) in Indonesia (Setianingrum et al., 2020). In cases of COPD exacerbation, *Aspergillus* IgG was detectable in 33% of 62 patients in France (Fréalle et al., 2021).

In India, ∼24% of 77 treated PTB patients had detectable *Aspergillus* antibody, without additional data to confidently support a diagnosis of CPA (Kurhade et al., 2002). Similar numbers of Japanese patients with active or treated PTB (19% of 226) had detectable *Aspergillus* antibody (Iwata et al., 1989), as did 9% of 101 HIV-positive patients completing pulmonary PTB treatment in Kampala (Kwizera et al., 2017). Our study did not use *Aspergillus* IgG antibody as the sole criterion for estimating CPA. Moreover, a CPA diagnosis in those with positive *Aspergillus* cultures in respiratory fluids required consistent radiology and symptoms (if an aspergilloma was not present) or histological confirmation after surgery. This definition of CPA also requires a minimum 3-month timeframe, which is important given the symptom overlap with other chest diseases.

Major uncertainty remains around several aspects of our estimations, in particular, the proportion of patients with dual infection at the beginning of ATT — our estimate is believed to be conservative. In those with confirmed PTB, the diagnosis of CPA is problematic given that radiological findings and symptoms overlap. Not enough is known about the antibody response to *Aspergillus* in this context, as a considerable proportion of patients have detectable IgG (and probably IgM and IgA) antibodies, which may or may not be reflective of dual infection. What is not known is whether dual infection may contribute to death, and require therapy for both infections, as has been well documented for NTM and *Aspergillus* dual infection (Jhun et al., 2020; Lowes et al., 2017).

CPA and drug-resistant tuberculosis have also been described, although infrequently. Most patients with MDR PTB have bilateral disease and extensive cavitation (Meghji et al., 2016), and dual infection with *Aspergillus* is anticipated. Too few data are available to estimate the frequency of CPA in MDR PTB. Furthermore, CPA may precede, occur as a coinfection with, or follow NTM infection (Jhun et al., 2020). NTM infection is one cause of smear- or GeneXpert-negative PTB, and may contribute to some of these misdiagnosed cases who also have CPA.

The mortality rate could be inflated by selective reporting of hospitalized cases or those at tertiary care centres, which would tend to see the worst cases, and by patients older than the usual age of tuberculosis onset in many parts of the world, including India. On the other hand, the lack of a CPA diagnosis, and either no treatment or mistreatment, is likely to enhance mortality. Our prior study in 2011 assumed a 15% annual death or surgical resection rate. For the present study this was adjusted to 20% in year 1 and 7.5% in years 2 to 5, reflecting the higher initial mortality from CPA, but better overall 5-year survival (from 75% mortality to 50%). Some data suggest a better survival rate for CPA complicating PTB (Koyama et al., 2015; Maitre et al., 2021), while other series do not (Lowes et al., 2017), potentially as a result of covariates such as male sex, diabetes, malnutrition, and age.

Additional published data on CPA occurring after recovery from PTB attests to the relatively high frequency of this complication. In Iran, 25 of 30 (83%) patients who returned to TB care had detectable *Aspergillus* antibodies, of whom 31% had CPA. Hedayati 2015 In Vietnam, 38 of 70 (54%) patients with recurrent symptoms after PTB cure had CPA; in India, 57% of 100 patients with recurrent symptoms after PTB had CPA (Nguyen et al., 2021; Singla et al., 2021). Only the first of these studies provided a total denominator, so these studies could not be used to inform our estimate of the post-ATT proportion with CPA.

Our current estimation is limited to 5 years after PTB, but from our clinical experience, and analogous to TB, the true mortality curve is probably of much longer duration (Dodd et al., 2021). One study of 19 patients with aspergilloma and severe hemoptysis from Brazil found that the risk of CPA after PTB extended for 40 years, with only ∼50% of the patients presenting in the 5 years after PTB treatment (Sapienza et al., 2015). In the South-Asian region, of which India is a part, there are estimated to be around 54 million people who have recovered from PTB, some of whom are at risk. Hence our estimate is probably an underestimate of the total CPA burden post-PTB.

Given these uncertainties, a range of ± 30% could be considered appropriate for our estimates. Our approach contributes to a partial explanation of the PTB-associated respiratory disease burden. Framing PTB and CPA as syndemic, as PTB and COVID are being considered (Trajman et al., 2022), seems particularly appropriate in vulnerable populations, where the kinds of case follow-up called for by Dodd et al. need to occur as part of PTB programs (Dodd et al., 2021). It is clearly unacceptable for a relevant cause of 40% of PTB-labelled deaths to remain unidentified, with the associated treatment possibilities based on greater diagnostic scrutiny.

## Appendix Supplementary materials

Image, application 1
