# Supplementary material for: New estimation of the prevalence of chronic pulmonary aspergillosis (CPA) related to pulmonary TB – a revised burden for India
Source: IJID Reg. 2022 Nov 18;6:7–14. doi: 10.1016/j.ijregi.2022.11.005 (PMC9772841; doi:10.1016/j.ijregi.2022.11.005)
Supplement: Supplementary file 1 [file mmc1.doc]

**Contents:**

Supplementary 1

1. Systematic review on prevalence and morbidity associated with CPA…………..…………… 1
2. PRISMA diagram ………………………………………………………………………………2-3
3. Table S1: Published studies of CPA with incidence and prevalence data ……...……….......... 4-12
4. Table S2. Published studies of CPA with survival data………………………………………..13-14
5. References (Studies in table S1 & S2)....……………………………………………………….15-17
6. Systematic review on prevalence and morbidity associated with CPA

Systematic review (SR) of the existing literature was done to search for relevant studies on prevalence and mortality associated with CPA. First Pubmed and Embase databases were searched for any existing systematic reviews which returned negative. We then screened the said databases for all studies on the said topic without any date or language restrictions up to June 2022.

Morbidity Search strategy: (((chronic) AND (aspergilloses, lung[MeSH Terms])) OR (CPA) OR (CCPA) OR (CNPA) OR (chronic pulmonary aspergillosis) OR (aspergillom*))) AND (incidence OR prevalence OR burden OR frequency).

Total articles identified: 532, After merging: 516 distinct papers were retrieved as well as 10 by data review. Of these, 22 were reviews, nine were case reports or small series, 419 did not meet inclusion criteria (including papers reporting only cultures of *Aspergillus* spp. from respiratory samples, unclear denominators or time frames with respect to ATT), seven were surgical series, 40 were studies not reporting original data and five duplicate data. Twenty four series were selected for inclusion. (Table 1)

Mortality search strategy –(((chronic) AND (aspergilloses, lung[MeSH Terms])) OR (CPA) OR (CCPA) OR (CNPA) OR (chronic pulmonary aspergillosis) OR (aspergillom*))) AND (mortality OR survival)

Total articles identified: 583. Papers not reporting data on chronic pulmonary aspergillosis (n=363), surgical series (n=87), reviews without original data (n=38), case reports and CPA series with <10 cases (n= 47) were excluded. Some CPA series reported no mortality data, were duplicate subset reports, autopsy series or did not conform to acceptable diagnostic criteria and we elected to not include series published over 50 years ago (given general changes in medical practice and society) and one study exclusively reported in patients with lung cancer. Twenty six series were selected for inclusion (Table 2).

Supplementary figure 1. PRISMA diagram of search for articles on incidence or prevalence of chronic pulmonary aspergillosis

(Records identified through electronic database) PUBMED, EMBASE

(N=532)

Records screened with abstract after removing duplication

(N=516)

Articles excluded (n=502)

Reasons for exclusion

Review papers, editorials, case reports

Did not meet inclusion criteria

Articles added by manual entry

(N=9)

Literature included in review (N= 23)

Supplementary figure 2. PRISMA diagram of search for articles on mortality of chronic pulmonary aspergillosis

**Mortality**

Records identified through electronic database (PUBMED)

(N=583)

Records screened with abstract after removing duplication

(N=561)

Articles excluded (n=535)

Reasons for exclusion

Not CPA

Surgical series

Reviews

Case reports and series with <10 cases (n=47)

Articles added by manual entry

(N=22)

Literature included in review (N= 26)

1. Table S1: Published studies of CPA with incidence and prevalence data

| **Sr No** | **Authors/State** | **Year of publication** | **Time period of estimation** | **Type of study** | **Adult/Pediatrics** | **Type of patients** | **Region** | **No of patients in study** | **Results/Prevalence/Burden/Frequency** | **others** |
| --- | --- | --- | --- | --- | --- | --- | --- | --- | --- | --- |
| **1** | Anonymous (Research committee of the British Tuberculosis Association)(Aspergillus in persistent lung cavities after tuberculosis. A report from the Research Committee of the British Tuberculosis Association, 1968) (Aspergilloma and residual tuberculous cavities--the results of a resurvey, 1970) | 1968, 1970 | First survey (1964-65), resurvey 3-4 years later | ? prospective | NA | Patients with post tuberculous cavities | 55 chest clinics in Great Britain | 544 patients in first survey; 417 in the second survey | First survey: Radiographic evidence of an aspergilloma was present in 11%  and of a probable aspergilloma in 3% of the total, whilst 10% had precipitins without  such radiographic appearances. Second survey: 34% of the survivors had precipitins. 17% had aspergillomas and 3% had probable aspergillomas with precipitins, and 14%; had precipitins alone. | - |
| **2** | Agarwal et al(R et al., 2012) | 2012 | January 2006 to December 2010 | Retrospective | Adults | Patients with ABPA & central bronchiectasis (CB) | Chandigarh | 179 patients | 8 (4.5%) out of 179 patients with ABPA-CB had Aspergilloma | ‘Aspergilloma’ was defined by the presence of eccentric soft tissue opacity within a bronchiectatic cavity |
| **3** | Nakamoto et al(K. Nakamoto et al., 2013) | 2013 | NA | Retrospective | ? Adults (Median age 70 years) | HIV negative patients who had *Mycobacterium*  *avium* complex lung disease (MAC-LD) | Saitama Cardiovascular and  Respiratory Center, Saitama, Japan, | 1079 patients | 23 (at time of diagnosis of MAC-LD) 19 (after diagnosis of MAC-LD) , 2.13% at time of diagnosis of MAC-LD) 1.8% (after diagnosis of MAC-LD) | Definition is not mentioned |
| **4** | Hedayati et al(Hedayati et al., 2015) | 2015 | March 2013  to March 2014) |  | Adults and Pediatrics (10-91 years) | 124 patients with TB (94 with  current TB and 30 with previous TB) | Masih Daneshvari Hospital in Tehran, Iran | 124 patients | 3 (2.4 %) met criteria for aspergilloma and 14 (11.3 %) for  CCPA. | Diagnosed according to Denning 2003 criteria; Clinical, radiological and precipitins levels |
| **5** | Oladele et al(Oladele et al., 2017) | 2017 | June 2014 – May 2015 | Cross-sectional Study | Mainly adults (Age 16-82) | HIV+ and HIV- patients at the end of their TB treatment | 3 centers in Nigeria (Lagos & Iloran) | 208 patients (HIV+:153, HIV:55) | 18, 8.7% (HIV+: 6.5%, HIV-:14.5%) | IgG cut off: 40 mg/L, Criteria: ESCMID/ERS 2016 |
| **6** | Kwizera et al(Kwizera et al., 2017) | 2017 | March 2013 and July 2014 | Nested cohort study under the prospective study “Study on  Outcomes related to Tuberculosis and HIV drug concentrations  in Uganda” (SOUTH) | Adults | All  participants were HIV-TB co-infected patients  with a diagnosis of their first episode of pulmonary  TB | Infectious  Diseases Institute, Kampala, Uganda | 101 patients | Aspergillus-specific IgG antibodies  were elevated in 4% (3/76) of HIV-infected Ugandan  adults at the start of TB treatment and in 9% (8/87) at the end of  TB treatment. Aspergillus-specific IgE antibody levels were measured at  end of TB treatment only and were elevated in  9.7% (9/93) | Only antibody levels were estimated.  A cut-off of 40 mg/l for the ImmunoCAP  Aspergillus-specific IgG levels as recommended by  manufacturer, and a cut-off of 20 mg/l for the Immulite  Aspergillus-specific IgG. A diagnostic cut-off of  <0.35kU/l for Aspergillus-specific IgE level |
| **7** | Munteanu et al(Munteanu et al., 2018) | 2018 | Dec2009 and Dec 2017 | NA | NA (median age of 55.7 ±14) | All patients referred with Bronchiectasis | Moldovan, National Bronchiectasis Centre | 334 patients | 14 (4.2%)  out 334 patients with bronchiectasis | Definition not specified |
| **8** | Page et al(Page et al., 2019) | 2019 | October 2012- January 2013 | community-based,  cross-sectional prospective survey | Age >16 years | Patients in the final month of pulmonary TB treatment or completed treatment  in 2005 or later | Gulu, Uganda | 398 patients initially assessed out of which 285 resurveyed | 4.9% (26% versus 0.8% in those with chest radiography cavitation versus those without. 3% versus 6.7% in HIV+ versus HIV-) . Annual rate of new CPA was 6.5% in those with cavitation and 0.2% in those without. | modified version of the DENNING et al. 2003  criteria was used. |
| **9** | Da Silva Matsuda et al(Matsuda et al., 2021) | 2020 | December 2012 to November 2013 | Cross-sectional study | Adults | Patients with history of cough, had provided two sputum samples that were smear negative (i.e., did not contain microscopically detectable acid-fast bacilli - AFB), and showed pulmonary radiological abnormalities | Two reference centres; Manaus, the capital city of Amazonas State, Brazil, | 213 patients | 10 (4.7%) out of 213 patients | Pulmonary mycosis was defined in accordance with the EORTC/MSG guidelines |
| **10** | Setianingrum et al(Setianingrum et al., 2020) | 2020 | February 2017 to February 2019 | prospective and cross- sectional study | Mainly adults | Patients  at the end of (4–6 months after starting) TB therapy. In  addition, healthy controls and diseased controls (patients with respiratory  symptoms but without a history or evidence of TB) were included | 6 centers in Indonesia | 203 patients | 26 out 203 patients, 24 (12%) with proven CPA and 2 (1%)  patients with probable CPA | Proven CPA was diagnosed based on the 3 parameters: 1) at least  one of these symptoms including cough, hemoptysis, chest pain, dyspnea,  fatigue, and/orweight loss ≥3months, AND 2) positive Aspergillus  IgG and/or positive Aspergillus spp. culture AND 3) radiological features  indicative of CPA (at least one of cavitation, fungal ball or pleural thickening).  Probable CPA was diagnosed in patients with all of: 1) at least  one of these symptoms including cough, hemoptysis, chest pain, dyspnea,  fatigue, and/or weight loss ≥3 months, 2) progressive cavitation  on serial chest radiographs, 3) negative TB PCR test for M. tuberculosis,  but without microbiological or serological confirmation of CPA |
| 11 | Kishan et al. (Kishan et al., 2020) | 2020 | NA | Observational study | Adults | Old treated TB patients | Ambala, Haryana, India | 110 patients | 39 (35.5%) out of 110 | Not immune compromised, with clinical signs and symptoms, Positive sputum and/or positive smear and/or  culture for Aspergillus and/or positive lung  histopathology suggestive of aspergillosis, suggestive chest imaging. |
| 12 | Lee et al(Lee et al., 2020) | 2020 | 2012-2019 | For active TB patients and TB close contact: cohort; control: group of healthy blood donors from  2012; old tb cases: cross sectional from follow-up in chest clinic between April, 2019 to June 2019 | adult (aged  >20 years old) | 4 groups; healthy volunteers, TB close contacts, active TB patients and old TB cases | Six hospitals in northern and  southern Taiwan | 1241 participants (including 200 healthy volunteers, 326 TB close contacts, 524 active  TB patients and 192 old TB cases). | 0.2% out of 524  active TB patient and 2.1% out of 192 old TB patients.  Using 27 mgA/L as cut-off level, the seropositive rate of A. fumigatus-specific IgG was 33.0% (66/200), 37.7% (123/326), 26.5% (139/524) and 43.2% (83/192) among the four groups, respectively | European Society for Clinical Microbiology  and Infectious Diseases (ESCMID)/European Respiratory  Society (ERS) and Infectious disease society of America (IDSA), 2016 criteria was used |
| 13 | Nguyen et al(Nguyen et al., 2021) | 2021 | October 2019 to December 2020 | Retrospective observational study | Adults  (22-86 years) | History of prior TB therapy who presented with abnormal radiological  findings and prolonged respiratory symptoms | Vietnam National Lung Hospital, Hanoi, Vietnam | 70 patients | 38 (54.3%) out of 70 patients | European Society for Clinical  Microbiology and Infectious Diseases and the European Respiratory Society criteria was used |
| 14 | Sehgal et al. (Sehgal et al., 2021) | 2021 | 1 January 2018 to 31 January 2020 | Prospective study | Adults (mean age: 42 years) | Clinically stable patients with non-CF bronchiectasis | Chandigarh | 196 patients | 196 (76%) had chronic aspergillus infection out of 258 patients | Chronic *A. fumigatus* infection defined as serum *A. fumigatus*-specific IgG  >27 mgA/L |
| 15 | Zubair et al(Zubair et al., 2021) | 2021 | January 2016 to December  2018. | Retrospective study | Adults (56 (SD±13.4) years | In patients treated for pulmonary TB (? at end of therapy) | Aga Khan University Hospital, Karachi, Pakistan | 115 patients | 17 (14.78%)  out of 115 patients | Definition is not mentioned |
| 16 | Singla et al(Singla et al., 2021) | 2021 | 1 February 2016-31 October 2016 | Prospective observational | Adults | Patients with post TB sequelae |  | 100 patients | 57 (57 %  ) out of 100 patients with post TB sequelae had CPA | Patients were classified as CPA if they met the four Global Action Fund for Fungal Infections criteria |
| 17 | Setianingrum et al(Setianingrum et al., 2021) | 2021 | February 2017 -April 2018 (baseline appointment) Patients were assessed at the start of TB therapy (baseline, 0–8  weeks) and at the end of TB therapy (at 5–6 months) | prospective, longitudinal cohort  study | Age > 16Years | All consecutive consenting patients with newly diagnosed  pulmonary TB | Indonesia (2 tertiary care hospital; 4 district hospital) | 216 patients recruited at baseline and 128 patients were re-evaluated at end of therapy | Incidence rate of proven and probable CPA at baseline were 12 (6%) and 5 (2%), and end of therapy 10 (8%) and 7 (5%) respectively. Aspergillus-specific  IgG was positive in 64 (30%) at start and became positive in 16 (13%) at end of therapy | Modified version of diagnosis of chronic pulmonary aspergillosis  in resource-limited  settings by Denning et al was used. |
| 18 | Kim et al(Kim et al., 2022) | 2022 | January 2015 and December 2018 | retrospective hospital based  cohort study | Adult | consecutive patients diagnosed with culture-  positive pulmonary tuberculosis | TB clinic of Hallym University Kangdong Sacred Heart Hospital (Seoul, South  Korea) | 345 patients | CPA developed in 10 (2.9%) patients at median 13.5 months (3.0-27.3) | European Society for Clinical Microbiology  and Infectious Diseases (ESCMID)/European Respiratory  Society (ERS) and Infectious disease society of America (IDSA), 2016 criteria was used |
| 19 | Namusobya et al(Namusobya et al., 2022) | 2022 | July 2020 and June 2021 | cross-sectional  study | Adults | microbiologically confirmed drug sensitive PTB (DS-PTB)  using  GeneXpert MTB/RIF and persisting pulmonary and/or systemic  symptoms despite 2 months of standard anti-TB  treatment | National TB control  center of Mulago National Referral Hospital (MNRH), Kampala,  Uganda, | 162 patients | 32 (19.8%) out of 162 patients | Global Fungal Infection Forum II diagnostic criteria for  CPA in resource-limited  settings was used |
| 20 | Oladele et al(Oladele et al., 2022) | 2022 | March 2016 to  February 2018. | longitudinal study | Adults | Confirmed (smear and/or  Xpert (PCR) and/or culture positive for Mycobacterium  tuberculosis) TB patients who were HIV- | National Institute of Medical Research and Lagos University  Teaching Hospital) in Lagos, Nigeria | Total 204 patients | Probable CPA in 10.4% at baseline, 15.1% at 3 months,  11.5% at 6 months, 16.7% at 9 months, and 19.3% at 12 months | Integrating the combination of persistent symptoms  (>3 months), CXR features of CPA and raised Aspergillus IgG  enabled a diagnosis of probable CPA at different time points |
| 21 | Irfan et al(Irfan et al., 2020) | 2022 | NA | retrospective cross-sectional study | NA | Smear negative pulmonary tuberculosis patients | tertiary care hospital in Karachi, Pakistan | 61 patients | 1 (1.6%) out of 61 patients | Definition is not mentioned |
| 22 | Chaves et al(Volpe-Chaves et al., 2022) | 2022 | February 2016 and  November 2019. | cross-sectional  analytical study | Adults | prior or active PTB | Maria Aparecida  Pedrossian University Hospital (MAPUH) outpatient clinic or admitted  to the Regional Hospital of Mato Grosso do Sul (RHMS) in  Campo Grande, Mato Grosso do Sul | 193 patients | 21 (10.9%) (global prevalence). <6 months 1.6%; 6-12 months 7.7%; 13-24 months 20%; 25-36 months 33.3%; 37-48 months 0%; 49-60 months 75%, >60 months 40.7% | European Society for Clinical Microbiology  and Infectious Diseases (ESCMID)/European Respiratory  Society (ERS) and Infectious disease society of America (IDSA), 2016 criteria was used |
| 23 | Toychiev et al(Toychiev et al., 2022) | 2022 | May 2020 to May 2021 | prospective observational study | Adult | Pulmonary Tuberculosis (140 with  smear-positive and 60 smear-negative) | Republican Specialized Research and Practical Medical  Center of Epidemiology, Microbiology, Infectious and  Parasitic Diseases and the Republican Specialized Research  and Practical Medical Centre of Tuberculosis and  Pulmonology, Tashkent, Uzbekistan | 200 patients | 13 (6.5%) of  200 patients with PTB met the criteria for chronic cavitary  pulmonary aspergillosis, | European Society for Clinical  Microbiology and Infectious Diseases/European Respiratory  Society and Infectious Disease Society of America criteria was used |

1. Table S2. Published studies of CPA with survival data.

| **Author, year** | **Country** | **Design** | **Population** | **Period of study (yrs)** | **Number of patients** |
| --- | --- | --- | --- | --- | --- |
| Jewkes, 1983(Jewkes et al., 1983) | UK | Retrospective, cohort | Tertiary care | 24 | 85 |
| Tomlinson, 1987(Tomlinson and Sahn, 1987) | USA | Retrospective, TB and sarcoidosis cohorts | Tertiary care | 10 | 28 |
| Rumbak, 1996(Rumbak et al., 1996) | USA | Retrospective | Tertiary care |  | 11 |
| Ueda, 2001(Ueda et al., 2001) | Japan | Retrospective | Tertiary care | 10 | 41 |
| Lee, 2009(Lee et al., 2009) | South Korea | Retrospective | Tertiary care | 10 | 105 |
| Nam, 2010(Nam et al., 2010) | South Korea | Retrospective, cohort | Tertiary care | 12 | 43 |
| Ohba, 2012(Ohba et al., 2012) | Japan | Retrospective, cohort | Tertiary care | 8 | 42 |
| Jhun, 2013(Jhun et al., 2013) | South Korea | Retrospective, cohort | Tertiary care | 4 | 70 |
| Nakamato, 2013(Keitaro Nakamoto et al., 2013) | Japan | Retrospective, cohort | Tertiary care | 13.3 | 194 |
| Sapienza, 2015(Hemoptysis due to fungus ball after tuberculosis: A series of 21 cases treated with hemostatic radiotherapy - PubMed, n.d.) | Brazil | Retrospective, referral cohort with haemoptysis | Tertiary care | 5 | 21 |
| Koyama, 2015(Koyama et al., 2015) | Japan | Retrospective, referral cohort comparing TB and emphysema | Tertiary care | Up to 8 years | 100 |
| Cuccheto, 2015(Cucchetto et al., 2015) |  | Retrospective, cohort | Tertiary care | 1 | 21 |
| Takeda, 2016(Takeda et al., 2016) | Japan | Retrospective, cohort | Tertiary care | 5.5 | 41 |
| Chan, 2016(Chan et al., 2016) | Hong Kong | Retrospective, cohort | Tertiary care | 10 | 29 |
| Lowes, 2017(Lowes et al., 2017) | UK | Retrospective, cohort | National centre | 20 | 387 |
| Uzunhan, 2017(Uzunhan et al., 2017) | France | Retrospective, sarcoidosis cohort | Tertiary centre | 7 | 65 |
| Jhun, 2017(Jhun et al., 2017) | South Korea | Retrospective, NTM cohort | Tertiary care | 5.5 | 41 |
| Naito, 2018(Naito et al., 2018) | Japan | Retrospective, cohort | Tertiary care | 6 | 62 |
| Bongomin, 2018(Bongomin et al., 2018) | UK | Retrospective, cohort | National centre | 1 | 206 |
| Furuuchi, 2018(Furuuchi et al., 2018) | Japan | Retrospective, NTM cohort | Tertiary care | 7 | 20 |
| Aguilar-Company, 2017(Aguilar-Company et al., 2019) | Spain | Retrospective, cohort | Tertiary care | 6 | 28 |
| Ando, 2019(Ando et al., 2019) | Japan | Retrospective, CPA cohort with haemoptysis | Tertiary | 5 | 41 |
| Akram, 2021(Akram et al., 2021) | Pakistan | Retrospective, cohort | Tertiary care | 3 | 218 |
| Kimura, 2021(Kimura et al., 2021) | Japan | Retrospective, cohort | Tertiary care | 5.3 | 264 |
| Maitre, 2021(Maitre et al., 2021) | France | Retrospective, cohort admitted to hospital | National records | 10 | 17,290 |
| Despois, 2022(Despois et al., 2022) | Australia | Retrospective, cohort | Tertiary care | 7 | 28 |

**4. References (Studies in table S1 & S2)**

Aguilar-Company J, Martín MT, Goterris-Bonet L, Martinez-Marti A, Sampol J, Roldán E, et al. Chronic pulmonary aspergillosis in a tertiary care centre in Spain: A retrospective, observational study. Mycoses 2019;62:765–72. https://doi.org/10.1111/myc.12950.

Akram W, Ejaz MB, Mallhi TH, Syed Sulaiman SAB, Khan AH. Clinical manifestations, associated risk factors and treatment outcomes of Chronic Pulmonary Aspergillosis (CPA): Experiences from a tertiary care hospital in Lahore, Pakistan. PloS One 2021;16:e0259766. https://doi.org/10.1371/journal.pone.0259766.

Ando T, Kawashima M, Masuda K, Takeda K, Okuda K, Suzuki J, et al. Exacerbation of chronic pulmonary aspergillosis was associated with a high rebleeding rate after bronchial artery embolization. Respir Investig 2019;57:260–7. https://doi.org/10.1016/j.resinv.2018.12.009.

Aspergilloma and residual tuberculous cavities--the results of a resurvey. Tubercle 1970;51:227–45.

Aspergillus in persistent lung cavities after tuberculosis. A report from the Research Committee of the British Tuberculosis Association. Tubercle 1968;49:1–11.

Bongomin F, Harris C, Hayes G, Kosmidis C, Denning DW. Twelve-month clinical outcomes of 206 patients with chronic pulmonary aspergillosis. PloS One 2018;13:e0193732. https://doi.org/10.1371/journal.pone.0193732.

Chan JF-W, Lau SK-P, Wong SC-Y, To KK-W, So SY-C, Leung SS-M, et al. A 10-year study reveals clinical and laboratory evidence for the “semi-invasive” properties of chronic pulmonary aspergillosis. Emerg Microbes Infect 2016;5:e37. https://doi.org/10.1038/emi.2016.31.

Cucchetto G, Cazzadori A, Conti M, Cascio GL, Braggio P, Concia E. Treatment of chronic pulmonary aspergillosis with voriconazole: review of a case series. Infection 2015;43:277–86. https://doi.org/10.1007/s15010-014-0711-4.

Despois O, Chen SC-A, Gilroy N, Jones M, Wu P, Beardsley J. Chronic Pulmonary Aspergillosis: Burden, Clinical Characteristics and Treatment Outcomes at a Large Australian Tertiary Hospital. J Fungi Basel Switz 2022;8:110. https://doi.org/10.3390/jof8020110.

Furuuchi K, Ito A, Hashimoto T, Kumagai S, Ishida T. Risk stratification for the development of chronic pulmonary aspergillosis in patients with Mycobacterium avium complex lung disease. J Infect Chemother Off J Jpn Soc Chemother 2018;24:654–9. https://doi.org/10.1016/j.jiac.2018.04.002.

Hedayati MT, Azimi Y, Droudinia A, Mousavi B, Khalilian A, Hedayati N, et al. Prevalence of chronic pulmonary aspergillosis in patients with tuberculosis from Iran. Eur J Clin Microbiol Infect Dis 2015;34:1759–65. https://doi.org/10.1007/s10096-015-2409-7.

Hemoptysis due to fungus ball after tuberculosis: A series of 21 cases treated with hemostatic radiotherapy - PubMed. n.d. https://pubmed.ncbi.nlm.nih.gov/26612361/ (accessed July 8, 2022).

Irfan M, Zahid A, Vaqar M, Sharif A, Jabeen K, Zubairi ABS. Alternate diagnosis in clinically diagnosed pulmonary tuberculosis patients treated at tertiary care hospital in a high TB burden country. Eur Respir J 2020;56. https://doi.org/10.1183/13993003.congress-2020.514.

Jewkes J, Kay PH, Paneth M, Citron KM. Pulmonary aspergilloma: analysis of prognosis in relation to haemoptysis and survey of treatment. Thorax 1983;38:572–8. https://doi.org/10.1136/thx.38.8.572.

Jhun BW, Jeon K, Eom JS, Lee JH, Suh GY, Kwon OJ, et al. Clinical characteristics and treatment outcomes of chronic pulmonary aspergillosis. Med Mycol 2013;51:811–7. https://doi.org/10.3109/13693786.2013.806826.

Jhun BW, Jung WJ, Hwang NY, Park HY, Jeon K, Kang E-S, et al. Risk factors for the development of chronic pulmonary aspergillosis in patients with nontuberculous mycobacterial lung disease. PloS One 2017;12:e0188716. https://doi.org/10.1371/journal.pone.0188716.

Kim C, Moon J-W, Park Y-B, Ko Y. Serological Changes in Anti-Aspergillus IgG Antibody and Development of Chronic Pulmonary Aspergillosis in Patients Treated for Pulmonary Tuberculosis. J Fungi Basel Switz 2022;8:130. https://doi.org/10.3390/jof8020130.

Kimura Y, Sasaki Y, Suzuki Junko, Suzuki Jun, Igei H, Suzukawa M, et al. Prognostic factors of chronic pulmonary aspergillosis: A retrospective cohort of 264 patients from Japan. PloS One 2021;16:e0249455. https://doi.org/10.1371/journal.pone.0249455.

Kishan J, Yadav A, Singhal S. To determine the previlance of fungal infection among patient with sputum negative old treated pulmonary tuberculosis. JK Sci 2020;22:80–3.

Koyama K, Ohshima N, Suzuki J, Kawashima M, Okuda K, Sato R, et al. Evaluation of clinical characteristics and prognosis of chronic pulmonary aspergillosis depending on the underlying lung diseases: Emphysema vs prior tuberculosis. J Infect Chemother Off J Jpn Soc Chemother 2015;21:795–801. https://doi.org/10.1016/j.jiac.2015.08.006.

Kwizera R, Parkes-Ratanshi R, Page ID, Sekaggya-Wiltshire C, Musaazi J, Fehr J, et al. Elevated Aspergillus-specific antibody levels among HIV infected Ugandans with pulmonary tuberculosis. BMC Pulm Med 2017;17. https://doi.org/10.1186/s12890-017-0500-9.

Lee JG, Lee CY, Park IK, Kim DJ, Chang J, Kim SK, et al. Pulmonary aspergilloma: analysis of prognosis in relation to symptoms and treatment. J Thorac Cardiovasc Surg 2009;138:820–5. https://doi.org/10.1016/j.jtcvs.2009.01.019.

Lee M-R, Huang H-L, Chen L-C, Yang H-C, Ko J-C, Cheng M-H, et al. Seroprevalence of Aspergillus IgG and disease prevalence of chronic pulmonary aspergillosis in a country with intermediate burden of tuberculosis: a prospective observational study. Clin Microbiol Infect Off Publ Eur Soc Clin Microbiol Infect Dis 2020;26:1091.e1-1091.e7. https://doi.org/10.1016/j.cmi.2019.12.009.

Lowes D, Al-Shair K, Newton PJ, Morris J, Harris C, Rautemaa-Richardson R, et al. Predictors of mortality in chronic pulmonary aspergillosis. Eur Respir J 2017;49:1601062. https://doi.org/10.1183/13993003.01062-2016.

Maitre T, Cottenet J, Godet C, Roussot A, Abdoul Carime N, Ok V, et al. Chronic pulmonary aspergillosis: prevalence, favouring pulmonary diseases and prognosis. Eur Respir J 2021;58:2003345. https://doi.org/10.1183/13993003.03345-2020.

Matsuda J da S, Wanke B, Balieiro AA da S, Santos CS da S, Cavalcante RCDS, Muniz M de M, et al. Prevalence of pulmonary mycoses in smear-negative patients with suspected tuberculosis in the Brazilian Amazon. Rev Iberoam Micol 2021;38:111–8. https://doi.org/10.1016/j.riam.2020.12.004.

Munteanu O, Volosciuc I, Scutaru E, Botnaru V. Chronic pulmonary aspergillosis in patients with post-tuberculosis bronchiectasis. Eur Respir J 2018;52. https://doi.org/10.1183/13993003.congress-2018.PA2743.

Naito M, Kurahara Y, Yoshida S, Ikegami N, Kobayashi T, Minomo S, et al. Prognosis of chronic pulmonary aspergillosis in patients with pulmonary non-tuberculous mycobacterial disease. Respir Investig 2018;56:326–31. https://doi.org/10.1016/j.resinv.2018.04.002.

Nakamoto Keitaro, Takayanagi N, Kanauchi T, Ishiguro T, Yanagisawa T, Sugita Y. Prognostic factors in 194 patients with chronic necrotizing pulmonary aspergillosis. Intern Med Tokyo Jpn 2013;52:727–34. https://doi.org/10.2169/internalmedicine.52.9142.

Nakamoto K., Takayanagi N, Ohkuma K, Gochi M, Kawate E, Yamakawa H, et al. Chronic pulmonary aspergillosis as a negative prognostic factor for patients with mycobacterium avium complex lung disease: Incidence and risk factors. Respirology 2013;18:105. https://doi.org/10.1111/resp.12184.

Nam H-S, Jeon K, Um S-W, Suh GY, Chung MP, Kim H, et al. Clinical characteristics and treatment outcomes of chronic necrotizing pulmonary aspergillosis: a review of 43 cases. Int J Infect Dis IJID Off Publ Int Soc Infect Dis 2010;14:e479-482. https://doi.org/10.1016/j.ijid.2009.07.011.

Namusobya M, Bongomin F, Mukisa J, Olwit WK, Batte C, Mukashyaka C, et al. Chronic pulmonary aspergillosis in patients with active pulmonary tuberculosis with persisting symptoms in Uganda. Mycoses 2022;65:625–34. https://doi.org/10.1111/myc.13444.

Nguyen NTB, Le Ngoc H, Nguyen NV, Dinh LV, Nguyen HV, Nguyen HT, et al. Chronic Pulmonary Aspergillosis Situation among Post Tuberculosis Patients in Vietnam: An Observational Study. J Fungi Basel Switz 2021;7:532. https://doi.org/10.3390/jof7070532.

Ohba H, Miwa S, Shirai M, Kanai M, Eifuku T, Suda T, et al. Clinical characteristics and prognosis of chronic pulmonary aspergillosis. Respir Med 2012;106:724–9. https://doi.org/10.1016/j.rmed.2012.01.014.

Oladele RO, Gbajabimiala T, Irurhe N, Skevington SM, Denning DW. Prospective Evaluation of Positivity Rates of Aspergillus-Specific IgG and Quality of Life in HIV-Negative Tuberculosis Patients in Lagos, Nigeria. Front Cell Infect Microbiol 2022;12. https://doi.org/10.3389/fcimb.2022.790134.

Oladele RO, Irurhe NK, Foden P, Akanmu AS, Gbaja-Biamila T, Nwosu A, et al. Chronic pulmonary aspergillosis as a cause of smear-negative TB and/or TB treatment failure in Nigerians. Int J Tuberc Lung Dis Off J Int Union Tuberc Lung Dis 2017;21:1056–61. https://doi.org/10.5588/ijtld.17.0060.

Page ID, Byanyima R, Hosmane S, Onyachi N, Opira C, Richardson M, et al. Chronic pulmonary aspergillosis commonly complicates treated pulmonary tuberculosis with residual cavitation. Eur Respir J 2019;53. https://doi.org/10.1183/13993003.01184-2018.

R A, An A, M G, B S, D G, A C. Allergic bronchopulmonary aspergillosis with aspergilloma: an immunologically severe disease with poor outcome. Mycopathologia 2012;174. https://doi.org/10.1007/s11046-012-9535-x.

Rumbak M, Kohler G, Eastrige C, Winer-Muram H, Gavant M. Topical treatment of life threatening haemoptysis from aspergillomas. Thorax 1996;51:253–5. https://doi.org/10.1136/thx.51.3.253.

Sehgal IS, Dhooria S, Prasad KT, Muthu V, Aggarwal AN, Rawat A, et al. Sensitization to A fumigatus in subjects with non-cystic fibrosis bronchiectasis. Mycoses 2021;64:412–9. https://doi.org/10.1111/myc.13229.

Setianingrum F, Rozaliyani A, Adawiyah R, Syam R, Tugiran M, Sari CYI, et al. A prospective longitudinal study of chronic pulmonary aspergillosis in pulmonary tuberculosis in Indonesia (APICAL). Thorax 2021. https://doi.org/10.1136/thoraxjnl-2020-216464.

Setianingrum F, Rozaliyani A, Syam R, Adawiyah R, Tugiran M, Sari CYI, et al. Evaluation and comparison of automated and manual ELISA for diagnosis of chronic pulmonary aspergillosis (CPA) in Indonesia. Diagn Microbiol Infect Dis 2020;98:115124. https://doi.org/10.1016/j.diagmicrobio.2020.115124.

Singla R, Singhal R, Rathore R, Gupta A, Sethi P, Myneedu VP, et al. Risk factors for chronic pulmonary aspergillosis in post-TB patients. Int J Tuberc Lung Dis Off J Int Union Tuberc Lung Dis 2021;25:324–6. https://doi.org/10.5588/ijtld.20.0735.

Takeda K, Imamura Y, Takazono T, Yoshida M, Ide S, Hirano K, et al. The risk factors for developing of chronic pulmonary aspergillosis in nontuberculous mycobacteria patients and clinical characteristics and outcomes in chronic pulmonary aspergillosis patients coinfected with nontuberculous mycobacteria. Med Mycol 2016;54:120–7. https://doi.org/10.1093/mmy/myv093.

Tomlinson JR, Sahn SA. Aspergilloma in sarcoid and tuberculosis. Chest 1987;92:505–8. https://doi.org/10.1378/chest.92.3.505.

Toychiev A, Belotserkovets V, Ignat’ev N, Madrakhimov S, Shaislamova M, Osipova S. Prevalence of chronic pulmonary aspergillosis and the antifungal drug resistance of Aspergillusspp. in pulmonary tuberculosis patients in Uzbekistan. Trop Doct 2022:494755221110678. https://doi.org/10.1177/00494755221110678.

Ueda H, Okabayashi K, Ondo K, Motohiro A. Analysis of various treatments for pulmonary aspergillomas. Surg Today 2001;31:768–73. https://doi.org/10.1007/s005950170045.

Uzunhan Y, Nunes H, Jeny F, Lacroix M, Brun S, Brillet P-Y, et al. Chronic pulmonary aspergillosis complicating sarcoidosis. Eur Respir J 2017;49:1602396. https://doi.org/10.1183/13993003.02396-2016.

Volpe-Chaves CE, Venturini J, B. Castilho S, S. O. Fonseca S, F. Nunes T, T. Cunha EA, et al. Prevalence of chronic pulmonary aspergillosis regarding time of tuberculosis diagnosis in Brazil. Mycoses 2022. https://doi.org/10.1111/myc.13465.

Zubair SM, Jabeen K, Irfan M. Frequency of chronic pulmonary aspergillosis in patients treated for pulmonary tuberculosis at a tertiary care hospital in Karachi, Pakistan. Eur Respir J 2021;58. https://doi.org/10.1183/13993003.congress-2021.PA1024.
